# Information from the Internet and the doctor-patient relationship: the patient perspective – a qualitative study

**DOI:** 10.1186/1471-2296-8-47

**Published:** 2007-08-16

**Authors:** Fiona A Stevenson, Cicely Kerr, Elizabeth Murray, Irwin Nazareth

**Affiliations:** 1 Department of Primary Care and Population Sciences, University College London, Hampstead Campus, Rowland Hill Street, London NW3 2PF, UK; 2 Department of Primary Care and Population Sciences, University College London, Archway Campus, Level 2 Holburn Union Building, Highgate Hill, London N19 5LW, UK

## Abstract

**Background:**

Both doctors and patients may perceive the Internet as a potential challenge to existing therapeutic relationships. Here we examine patients' views of the effect of the Internet on their relationship with doctors.

**Methods:**

We ran 8 disease specific focus groups of between 2 and 8 respondents comprising adult patients with diabetes mellitus, ischaemic heart disease or hepatitis C.

**Results:**

Data are presented on (i) the perceived benefits and (ii) limitations of the Internet in the context of the doctor-patient relationship, (iii) views on sharing information with doctors, and (iv) the potential of the Internet for the future. Information from the Internet was particularly valued in relation to experiential knowledge.

**Conclusion:**

Despite evidence of increasing patient activism in seeking information and the potential to challenge the position of the doctor, the accounts here do not in any way suggest a desire to disrupt the existing balance of power, or roles, in the consultation. Patients appear to see the Internet as an additional resource to support existing and valued relationships with their doctors. Doctors therefore need not feel challenged or threatened when patients bring health information from the Internet to a consultation, rather they should see it as an attempt on the part of the patient to work with the doctor and respond positively.

## Background

Recent advances in information technology, such as the Internet, touch-screen public kiosks, and cable television have revolutionised access to health information [1]. In the UK various Departments of Health initiatives have emphasised the need to give greater voice and influence to users of NHS services, and patients are expected to assume greater responsibility for their personal health [2,3]. This both mirrors and fuels a demand for more and better health information.

Information accessed via the Internet crosses international boundaries [4] and includes access to experiential knowledge from other patients and carers, enabling patients to become more active collaborators in their own health [5].

It has been argued that advances in information technology fundamentally challenge the very essence of doctor-patient consultations by "substituting impersonal exchanges across luminescent LCD screens for the face-to-face encounters and hands-on care that produce much of the therapeutic benefit and professional satisfaction of primary care practice"[6].

This somewhat simplistic and apocalyptic vision is however brought into doubt by the research evidence. A study conducted in the USA found that the effect of a patient taking health information from the Internet into a consultation was likely to be positive so long as the physician has adequate communication skills [7], with a perceived deterioration in the doctor-patient relationship being much more likely if the doctor felt or acted challenged [8].

From the patient perspective, a quantitative study conducted in Australia concluded that the majority of patients did not believe that information searching adversely affected the doctor-patient relationship [9], while a parallel study with doctors found they were broadly supportive of patients searching the Internet for medical information [10].

However despite the seemingly positive comments about patients' use of the Internet the extent to which patients discuss the results of searches for information with health care professionals is unclear. This may be because searches may not directly raise issues but rather inform existing communication and decisions, and therefore patients may not feel the need to discuss the information they have found [11]. The qualitative work of Henwood et al [12] and Broom [13] suggested that people may fear overstepping the role of patient and appearing to tell the doctor their job, or alternatively may feel that doctors need to be protected from the 'informed patient' who may exert extra pressure on an already busy professional.

In summary, neither the immediate impact nor future implications of the Internet for the ways in which patients access healthcare and their relationship with professionals is yet clear [14]. Studies suggest that the medical profession are still perceived to be the most important source of health information [11,12,15,16], and that information gathered online may complement rather than oppose information delivered by medical professionals [17]. However few researchers have attempted to address how this new medium influences patients' perceptions of encounters with medical professionals [13].

The findings presented here originate from a study utilising a focus group technique in which it was possible to both explore and discuss patients' thoughts and opinions as to how they handled information from sources such as the Internet and their views on the effect of this on their relationship with their doctor. The responses were not directly sought but rather volunteered as part of a discussion about the usefulness or otherwise of three Internet interventions used prior to the focus group. Although prompted by immediate prior use of Internet interventions, by not asking directly about perceptions of the effect of the Internet on their relationship with medical practitioners the accounts obtained are less likely to be a socially constructed response. In other words respondents will be less likely to have constructed their responses with the aim of 'pleasing' the researchers by providing what they think might be the expected response.

## Methods

The data originate from a study to determine patients' requirements of Internet interventions and the criteria they used for assessing such applications[18]. Respondents used three Internet interventions and then took part in a disease specific focus group to examine their thoughts and reactions. Focus groups were used to capitalise on the interaction between respondents. We consulted consumer representatives on the design of the study, topic guide, study procedures, analysis and emerging findings. Ethical approval was obtained from the relevant local research ethics committees. Details of the findings relating to requirements for Internet interventions are available elsewhere [19].

### Sampling and recruitment

The sample comprised 34 adult patients with diabetes mellitus, ischaemic heart disease or hepatitis C. These are all common chronic conditions that have been designated as national health priorities and included at least one condition that patients may experience as stigmatising. The research was conducted in three UK areas varying in socio-economic and ethnic diversity in order to include as wide a range of views as possible and thereby increase the potential for the transferability of the findings. Recruitment was carried out in both clinical and community settings. People were invited to take part through advertisements in local newspapers and patient newsletters, posters in general practice clinics and flyers given out in patient self-help group meetings, exercise classes, and hospital outpatient clinics. The characteristics of the participants are shown in Table 1.

**Table 1:** Self-reported characteristics of 34 patient participants

		**Number of participants (%)**
Gender	Male	22 (64.7)
	Female	12 (35.3)
Age (years)	30 – 39	3 (8.8)
	40 – 49	4 (11.8)
	50 – 59	7 (20.6)
	60 – 69	16 (47.0)
	70 – 79	4 (11.8)
Employment	Employed	8 (23.5)
	Economically inactive	26 (76.5)
Education	School leaver	8 (23.5)
	A levels or vocational equivalent	7 (20.6)
	Degree, HND or similar	17 (50.0)
	Undisclosed	2 (5.9)
Ethnic group	White British	26 (76.5)
	White European (non-British origin)	5 (14.7)
	Asian or British Asian	2 (5.9)
	Black or Black British	1 (2.9)
English first language	Yes	29 (85.3
	No	5 (14.7)
Computer experience	Novice	4 (11.8)
	Basic	9 (26.5)
	Experienced	21 (61.7)
Time since diagnosis (years)	Less than 1	4 (11.8)
	Between 1 and 5	8 (23.5)
	More than 5	18 (52.9)
	Undisclosed	4 (11.8)

Wherever possible in arranging the groups we sought diversity in relation to level of computer experience, time since diagnosis and educational qualifications.

### Data collection

Three Internet interventions were chosen for each condition. Suitable interventions were identified through authors of studies reporting on the development and/or evaluation of such interventions in the academic literature, Google Internet searches for each of the relevant long-term conditions and by asking researchers, academics, and consumer representatives for interventions known to them personally. Interventions that only provided health information with no interactive components were excluded, as were those aimed at more than one condition. We chose interventions made in different countries and developed by different stake-holders (commercial, academic, medical) with varied approaches to information presentation. Details of the actual interventions used are presented in Table 2.

**Table 2:** Details of Internet Interventions shown to participants

Condition	Internet intervention	Further details
Patients with diabetes mellitus	My diabetes Freely available at	Identified by google internet search Produced by US commercial stake-holder
	Diabetes insight Freely available at	Identified by google internet search Produced by UK charitable stake-holder
	Aida Freely available at	Identified via systematic review search of academic literature Produced by European academic stake-holders
Patients with ischaemic heart disease	Heart center online Freely available at	Identified by google internet search Produced by US commercial stake-holder
	Your heart Freely available at	Identified by google internet search Produced by UK NHS and academic stakeholders
	Heart info Freely available at	Identified by google internet search Produced by US charitable stake-holder
Patients with hepatitis C	Hepatitis C forum Freely available at	Identified by consumer representative Produced by German forum (stakeholder unclear); English version no longer available.
	Hep C vets Freely available at	Identified by consumer representative Produced by a US consumer representative stake-holder
	Hep C UK Freely available at	Identified by consumer representative Produced by UK charitable consumer representative stake-holder

### Focus groups

We ran 8 disease specific groups of between 2 and 8 respondents at community IT facilities. Details of each group are presented in Table 3. Group members were each allocated a computer with three Internet interventions appropriate to their condition bookmarked. They were asked to spend up to 30 minutes on each intervention before moving on to the next. There was a range of computer experience and facilitators provided help as necessary. Most respondents were able to navigate around the interventions provided with minimal or no help. After a short refreshment break respondents took part in a 90-minute focus group discussion that was audio-taped and transcribed. Given the health status of our respondents and the fact the research lasted half a day we were anxious not to tire them. Respondents were free to take a break from the computer at any time and refreshments were available throughout. We were careful to monitor respondents and stressed they were free to leave at any time. We encountered no problems and no respondents left before the end.

**Table 3:** Composition of focus groups

**Condition**	**Location**	**Number of participants**
Diabetes mellitus	London	2
Diabetes mellitus	London	2
Diabetes mellitus	London	3
Diabetes mellitus	Nottingham	6
Ischaemic heart disease	London	3
Ischaemic heart disease	London	8
Ischaemic heart disease	Exeter	6
Hepatitis C	London	4

Using a focus group technique enabled both agreements and disagreements to emerge. All disagreements were resolved within the groups without the need for intervention on the part of the facilitators. Both the agreements and disagreements enhanced the depth of the data obtained.

### Topic guide

The topic guide was developed following a review of the literature and discussion with consumer representatives and researchers in the field. No modification was needed after piloting. Areas covered included respondents' overall reactions to the Internet intervention used, preferences for, or dislike of, a particular programme and reasons for this, when and where similar programmes might be used, areas or information looked for but not found, reactions to scientific uncertainty, and any other comments.

### Qualitative analysis

Three members of the team (a health psychologist, a medical sociologist, and a primary care professional) developed an initial coding frame. This and the subsequent analysis was then discussed and developed within the whole team, which contained another primary care professional and a representative of one of the included patient groups. No computer software package was used to organise the analysis.

## Results

The four themes that emerged were: (i) the perceived benefits of the Internet in the context of the therapeutic relationship (ii) the limitations of the Internet (iii) views on sharing information obtained from the Internet with doctors and (iv) the potential of the Internet for the future.

In the findings that follow a code is used prior to the extracts to indicate the person who is speaking and the group they were part of. The letter 'R' is used to indicate speech by the researcher.

### Perceived benefits of the Internet

Information from the Internet was generally presented as supplementing that received in consultations and therefore supporting the therapeutic relationship. Given time limited consultations, the Internet was perceived to be particularly useful for confirming and expanding on information received without 'bothering' the doctor. This is explored in the following extract.

NADHD01 Well yes as this gentleman says sometimes you go to the doctor and when you come out you really don't know what it's all about. If you have access [to the Internet] and you can ask the questions what is it, how is this caused, how is it managed – basic questions and then little sites that you can click off on there to obtain a little bit more information if you wish.

(Diabetes group 4)

Accessing information via the Internet means that it is possible to confirm information that is already 'known' and revisit information.

LHDO6 ... it is back-up information from what we already know. And it's there permanently, whether it's on the screen or downloaded.

(Heart disease group 1)

Websites that provided lists of possible questions to ask the doctor were said to help to make the most of long awaited and time limited consultations, increasing the potential for patient involvement:

LHD07 There was one page on it which was questions to ask your doctor which was one of the things I went looking for and I found somewhere else had represented it much better but as an idea it was really good. Especially when you go to see a consultant and you wait months and months for that sort of thing to happen and your mind is full of all sorts of stuff. It's nice to go in with a written list or prepared list of things to ask.

(Heart disease group 2)

The Internet provides easy access to medical information outside of the consultation. One informant explained how he had used the Internet to search for information about a particular drug so as to engage with the doctor about a previous prescribing decision. This did not appear to be indicative of a lack of faith in, or attempt to undermine, the doctor, but rather a desire to have an informed discussion.

EHD02 Something I've discovered recently for instance is – my mother is on a drug called Seroxat which has been in the papers a lot recently because of the bad side effects and she does have side effects and I'm, I personally am convinced that it's because of this particular drug. Well I was able to look up all the details about that you know, the whys and the wherefores and where it's banned and where it's not banned and then you know we can see the doctor about it and say well, should she, are you quite sure?

(Heart disease group 3)

There was a general awareness on the part of respondents that there were limits to their GP's knowledge. For this reason respondents felt they had a responsibility to search for information.

NAD05 ... I've had to do a lot for meself to find out different things and you know the doctors are not even as much aware. I mean they can't all be experts in the one thing

(Diabetes group 4)

Respondents with Hepatitis C particularly expressed the view that GPs might not be well informed about their condition.

LHCO2 I asked my doctor, my GP a question about Hepatitis C and he quite openly said: 'I have no idea'.

(Hepatitis C group)

Practical advice and experiential knowledge relating to the day-to-day management of conditions and medical procedures was perceived by all groups to be very useful; beyond the doctor's expertise, but nevertheless easily accessible via the Internet.

LHD07 ... it's nice to know that whatever you've gone through or going through that people have been there beforehand and they've picked up information on the way. So that rather than reinventing the wheel it's sharing with other people and there are tips and tricks that you get from them that you just wouldn't get from a GP. Just little practical things.

(Heart disease group 2)

Finally, the ability to access a range of medical opinions, as well as options for medical treatment from other parts of the UK and/or countries was perceived to be useful, as it was suggested that the financial constraints of the NHS might restrict the options presented in consultations.

LHCO3 .... Most people believe that there is some kind of Government, you know, shut your mouth – you know what I mean – it's kind of like the government are keeping it [treatments not available on the NHS] secret because of the impact on the health service – I mean it would devastate the health service if -

LHCO4 Too expensive

LHCO2 Exactly

(Hepatitis C group)

### Perceived limitations of the Internet

Even where there was the facility to consult a medical professional over the Internet, it was felt that any advice provided was limited by the fact that it was not based on the individual's medical records and knowledge of their past medical history.

LHD07 Any doctor or GP you ask over the Internet is going to be so, have such limited information they would be very reticent to give you opinions or advice. Their first thing would be to go to your GP or go to your doctor because they've got your full case notes. So if they could get over that barrier it would be useful but it's a big barrier to get over.

R The fact that they wouldn't have information about you?

LHD07 It's like these dial in things you hear on the radio, these sort of radio doctors and telephone doctors you hear, you know 99 times out of 100 what they say is go to your GP.

(Heart disease group 2)

A particular concern was that information obtained via the Internet may not be considered as trustworthy as advice obtained directly from their doctor.

LHD06 I wouldn't trust a computer that much ... any specific information like 'do this' or 'don't do that', because – even though it may be useful, I'd much rather deal with a human being, a doctor

(Heart disease group 1)

In addition, having to write queries as opposed to verbally express them was presented as a possible barrier in terms of the level of difficulty involved in doing so.

EHD04 Yeah and are people articulate enough to be able to put down their question properly in the first place as against speaking it they have got to write it haven't they?

(Heart disease group 3)

### Sharing information from the Internet

There were reports that doctors positively encouraged people to search for information about their medical problems and potential treatment options.

LAD02 ... cos sometimes doctors just say to you that: 'Well, I'm going to put you on such-and-such', and recently some of the consultants I've been seeing have said to me: 'Are you Internet friendly – so do you want to go and look this up and then come back and talk to me about it?'

(Diabetes group 3)

Conversely, resistance from doctors to patients bringing information even about their day to day management into the consultation was not only anticipated but also experienced.

R ... say you were doing your blood pressure at home. Which a lot of people do do, then you could put that data into the computer, and the doctor would have that when you see them

LHD06 What your own doctor do you mean?

R Either your GP or your hospital doctor, you could choose

LHD10 I think doctors tend to get annoyed when you start telling them your own self-diagnosis.

(Heart disease group 1)

Respondents generally presented the Internet as a resource that supported and enhanced as opposed to challenged the therapeutic relationship and in keeping with this it was suggested that information found should be checked with the doctor.

LHDO5 As a sensible person ... you should go and check it out with your GP

(Heart group 1)

One of the respondents felt accessing the Internet for information was unnecessary as you could just ask your doctor. Other respondents in the group challenged this, but the fact it was raised demonstrates that not every patient believes the Internet is a useful resource in this context.

EHD04 ... I mean why should a person write a question of a medical nature, a medical enquiry when all they've got to do is go to their own surgery and ask the doctor.

EHD05 Well there are lots of reasons why they would do that, lots of reasons.

(Heart disease group 3)

### Future uses of the Internet in health care

Respondents suggested that a possible future use of Internet technology, saving time for both patients and practitioners, was performing automated tasks such as checking if test results are available, or using test results as a basis for informing people of the necessity for an appointment. It was also suggested that ongoing monitoring could be provided over the Internet. There was particular exploration of this by the diabetes groups.

R How would the website then give you an idea of how things were going?

LAD02 Because if I'm diarising over a period of time, it would give you feedback it looks like from the programme, on basically that you would be able to look and to read about how you've done this week, how you've done next week when it's put it all together, 'cos as a process it will do it for you, which is really good and really helpful. As opposed to you taking paper and pen and doing it, and you forget and then you – 'cos I can't remember at the end of the day everything that I've eaten, or whatever it is, and what I've done completely, you know, 'cos my brain's tired and you know, I'm just absolutely just at the end of the tab, and it takes too much. But if I had the programme, and I could just put in, dot in these true things as I went along, at the end of the week it's doing the work for me, calculating and saying: 'You've taken so much exercise, you've had so much insulin, this is what your calorie intake was, or whatever it was, and this is how much', you know – it would help, it would help, I think also it would give you some kind of incentive as well, so as to progress en route. And you could look, and it says on it in printout, so that you could take it to your medical practitioner at the end -

LAD04 Yes, that would be a really good idea.

LAD02 And that would be really useful because then they're sitting there going to you: 'So, how have you got on, and what did you do in the first' – 'I can't remember, I haven't been here for the last three months, or whatever, and you're asking me what did I do for the last three months', you know, and really and truly, I mean half the time when I go to the dietician I know that I'm making up fairytales, right, because I can't remember what I've eaten – I know that I've eaten, yeah, and I've tried some of the things that you told me, but you know, what time I'm eating, and when I'm eating, I can't -

(Diabetes group 3)

## Discussion

The Internet was perceived to be a useful resource given the limited time available in consultations, and was particularly valued for the breadth of information and the ease of access to experiential knowledge. It has been suggested that the Internet may alter the traditional imbalances in knowledge and power in the doctor-patient relationship [13,20], however our data indicate that the effect is more complex. Rather we suggest that information obtained via the Internet supports existing therapeutic relationships. The few accounts of searches that might contradict information from the consultation were not presented as attempts to challenge previous encounters but rather to clarify information provided or treatment decisions. Crucially it is clear that patients viewed seeking information from the Internet as fundamentally different from gaining information from a face to face encounter with their doctor. This was attributed to factors such as access to a full medical history and the humanity of dealing with a person face to face. This may have implications for the future direction and development of Internet interventions.

There has been a great deal of attention in the literature concerning patient activism in the consultation. There was some discussion and acceptance of the idea that doctors cannot be experts in everything and that patients therefore need to take some responsibility for finding out about their particular problems. It is, however, worth noting that activism outside of the consultation is lower risk in terms of upsetting the rules of interaction in consultations and therefore is arguably an attractive option for patients. The Internet was perceived to be particularly valuable in this endeavour as information could be easily accessed as necessary.

There was some interest in the potential of the Internet for services such as automated test results and routine monitoring. Yet this has to be considered alongside concerns raised about bringing information into the consultation. The Internet is unlikely to become an openly integrated part of medical interaction until both patients and practitioners can agree on the role the patient, in relation to introducing information into the consultation be it from the news media, books, or the Internet, should play and practitioners make it clear to patients that both the use, and discussion of the use, of information sources from outside the consultation are legitimate.

## Conclusion

Although the literature identifies possible tensions that may be caused by patients' access to information via the Internet, generally the Internet was perceived as a support, as opposed to a challenge, to medical practice. There was a continuing and strong awareness of the value of medical expertise. The accounts here do not in any way suggest a desire to disrupt the existing balance of power, or roles, in the consultation.

### Limitations

Our data are limited to people who were suffering from a chronic condition. Moreover, all of the included conditions required regular contact with the medical profession, and were either potentially fatal or had potentially fatal complications. For this reason the general faith placed in the medical profession is unsurprising, and cannot necessarily be said to be generalisable for other groups in the population.

It is also important to note that these data are people's accounts of how they use the Internet, and in creating these accounts they drew upon implicit rules and shared assumptions about a sensible and appropriate use of resources. As Nettleton et al [4] previously pointed out, the discursive repertoires that people so readily draw upon reveal as much about the prevailing discourses of professionalism, biomedicine and active citizenship as they do about the views and preferences of the actors themselves.

### Implications for practice

The effect of the availability of health related information on relationships with doctors is a central concern for patients. The sheer quantity and convenience of health information from the Internet facilitates people taking an active role in their care however in this study respondents generally reported attempts to manage use of the Internet so it caused minimal disruption to existing relationships in consultations. Therefore, doctors need not feel challenged or threatened when patients bring health information from the Internet to a consultation, rather they should see it as an attempt on the part of the patient to work with the doctor and respond positively.

## Competing interests

The authors declare that they have no competing interests.

## Authors' contributions

FS helped to refine the initial design, helped to collect, analyse and interpret the data and drafted the manuscript. CK helped to refine the initial design, led on collecting, analysing and interpreting the data and helped to draft the manuscript. EM initiated the study, helped to refine the initial design, helped to collect, analyse and interpret the data and helped to draft the manuscript. IN helped to refine the initial design, commented on analysis and interpretation of the data and helped to draft the manuscript. All authors read and approved the final manuscript.

## Pre-publication history

The pre-publication history for this paper can be accessed here:


